# Impact of Different Normality Thresholds for 24-hour ABPM at the Primary Health Care Level

**DOI:** 10.5935/abc.20160204

**Published:** 2017-02

**Authors:** Guilherme Brasil Grezzana, David William Moraes, Airton Tetelbon Stein, Lucia Campos Pellanda

**Affiliations:** 1 Instituto de Cardiologia - Fundação Universitária de Cardiologia; Porto Alegre, RS - Brazil; 2 Universidade Federal de Ciências da Saúde de Porto Alegre - UFCSPA; Porto Alegre, RS - Brazil; 3 Universidade Luterana do Brasil (ULBRA), Canoas, RS - Brazil

**Keywords:** Hypertension, Risk Factors, Blood Pressure Monitoring, Ambulatory, Primary Health Care, Antihypertensive Agents

## Abstract

**Background:**

Hypertension is an important risk factor for cardiovascular outcomes. Primary health care (PHC) physicians should be prepared to act appropriately in the prevention of cardiovascular risk factors. However, the rates of patients with control of blood pressure (BP) remain low. The impact of the reclassification of high BP by 24-hour ambulatory BP monitoring (ABPM) can lead to different medical decisions in PHC.

**Objective:**

To evaluate the agreement between the BP measured by a conventional method by PHC physicians and by 24-hour ABPM, considering different BP normal thresholds for the 24-hour ABPM according to the V Brazilian ABPM Guidelines and the European Society of Hypertension Guidelines.

**Methods:**

A cross-sectional study including 569 hypertensive patients. The BP was initially measured by the PHC physicians and, later, by 24-hour ABPM. The BP measurements were obtained independently between the two methods. The therapeutic targets for the conventional BP followed the guidelines by the Eighth Joint National Committee (JNC 8), the V ABPM Brazilian Guidelines, and the 2013 European Hypertension Guidelines.

**Results:**

There was an accuracy of 54.8% (95% confidence interval [95%CI] 0.51 - 0.58%) for the BP measured with the conventional method when compared with the 24-hour ABPM, with a sensitivity of 85% (95%CI 80.8 - 88.6%), specificity of 31.9% (95%CI 28.7 - 34.7%), and kappa value of 0.155, when considering the European Hypertension Guidelines. When using more stringent thresholds to characterize the BP as "normal" by ABPM, the accuracy was 45% (95%CI 0.41 - 0.47%) for conventional measurement when compared with 24-hour ABPM, with a sensitivity of 86.7% (95%CI 0.81 - 0.91%), specificity of 29% (95%CI 0.26 - 0.30%), and kappa value of 0.103.

**Conclusion:**

The BP measurements obtained by PHC physicians showed low accuracy when compared with those obtained by 24-hour ABPM, regardless of the threshold set by the different guidelines.

## Introduction

Hypertension, a chronic disease with an estimated prevalence of 40-45% in adults, is a recognized public health problem and the main cause of general mortality,^1^ for which low rates of control still remain.^2^ The hypertensive patient requires periodic medical care associated with adequate pharmacological therapy and lifelong lifestyle changes.^3^ The contribution of 24-hour blood pressure (BP) assessment by ambulatory BP monitoring (ABPM) in the diagnosis, monitoring, and prognostic stratification of hypertension is clearly defined in the literature.^4^

A national study compared the BP classification according to new thresholds established by the VI Brazilian Guideline for Hypertension / V Brazilian Guideline for ABPM, in relation to those previously established by the IV Brazilian Guideline for ABPM.^5^ The authors observed that the new thresholds substantially reclassified hypertension, increasing the percentage of hypertensive patients, especially for the variable systolic BP during sleep.^5^ When considering different thresholds for 24-hour borderline BP / hypertension and normal BP / borderline BP, and the reclassification of hypertensive patients in regard to their control, it was observed that the samples of patients in the studies were similar in regard to antihypertensive treatment. Regarding the IDACO study, ^6^ the guidelines from the European Society of Cardiology (ESC) have maintained BP thresholds for the definition of hypertension by 24-hour ABPM as values greater than or equal to 130 mmHg for systolic BP and 80 mmHg for diastolic BP.^7^

Two questions of application in clinical practice regarding the care of the hypertensive patient remain little explored: the applicability and the importance of more rigorous thresholds as criteria of normality for BP, and the prospective evaluation of the diagnostic accuracy between the different methods of BP measurement.^8^ These inquiries arise from a need for prospective studies and elaboration of databases, preferably national ones, to allow a more adequate assessment of the ABPM normality thresholds for the hypertensive population, especially those attended by the primary health care (PHC) system.

This study proposed to evaluate the diagnostic accuracy of BP measurement by the conventional method, performed by PHC physicians, and the agreement of these measures with those obtained by the 24-hour ABPM, set as normality thresholds by the guidelines.

## Methods

### Delineation and participants

The participants of this cross-sectional study conducted at two health posts included hypertensive patients from Antônio Prado (RS), a city in the south of Brazil with 12,883 inhabitants.^9^ All patients were hypertensive, were enrolled in the *Programa de Saúde da Família* (Family Health Program, PSF), took part in regular clinical follow-up at the hypertension outpatient clinic of the city's two *Unidades Básicas de Saúde* (Basic Health Units, UBS), and were on antihypertensive treatment for at least 6 months.

The samples were randomly selected from the total set of hypertensive patients enrolled in the UBSs, through random numbers generated by the program Microsoft Excel 2010. Patients were assessed by their own PHC physicians during routine visits to the hypertension outpatient clinic from January 2013 to October 2014. Patients who had participated in a previous cross-sectional study,^8^ which included an assessment with 24-hour ABPM, were contacted by phone and/or letter to participate in this new study. Those who were not able to answer the questionnaire, pregnant women, individuals with a non-sinus rhythm electrocardiogram, residents from outside the coverage area of the UBSs, patients who switched cities or who were not found, and those who did not tolerate the use of ABPM or who presented some technical difficulty in the application of the method were excluded. Thus, out of the total of 639 patients, 28 were excluded from the study due to complications related to technical problems in the reading and failures in the adjustment of the ABPM cuff (18 patients), sleep disorders that prevented adequate BP measurements (8 patients), and intolerance of the equipment due to anxiety (2 patients).

All subjects agreed to participate in the study and signed an informed consent form. The results of the biochemical tests and ABPM performed during the study were delivered to the patients. The project was approved by the Research Ethics Committee (REC) of the IC/FUC - 4278.08.

### Measurements performed

The BP was verified by the PHC physicians through three measurements with a mercury sphygmomanometer (the use of a mercury column sphygmomanometer is allowed in the state of Rio Grande do Sul, which follows the guidelines of the Resolution of the Collegiate Board of Directors no. 63, dated November 25, 2011, article 23) with orientation for individualized adjustment of the cuff, with the patients in a seated position and their feet resting on the floor and after a minimum rest period of 5 minutes. The physicians at the health centers were instructed to measure the BP in both arms, taking, as a reference, the highest value obtained after an approximate interval of 3 minutes between measurements. The first measurement was discarded, and the mean of the two subsequent measurements was calculated and recorded on the patient's chart. Then, during the same visit, the patient was referred to a nurse trained for this study who placed the device for the 24-hour ABPM, applied a standardized questionnaire, and obtained the anthropometric measurements. Subsequently, the patient's medical records were reviewed, biochemical exams were requested, and the ABPM report was prepared blindly by the investigating physician. The ABPM device was applied during a normal day of the patient's work activity, excluding days on the weekends and holidays. Based on prognostic evidence, the ABPM was selected as the standard reference for BP measurements and for assessing the diagnostic accuracy of the conventional BP measurement.^10^

The ABPM monitors used in the study were duly validated and calibrated according to international recommendations.^11^ The ABPM recorder used was the DMS Brasil model TM 2430 and the model of the mercury sphygmomanometer was the MDF 800. The ABPM was scheduled to record a BP measurement every 15 minutes during the waking period and every 30 minutes during sleep. The schedules were adjusted according to the individualization of the sleep and awakening habits of each patient. The obtaining of data from at least 60 records in the 24-hour period was considered adequate, with at least two records every hour during the sleep period. The parameters assessed by the ABPM were the mean systolic and diastolic BP from the 24-hour, waking, and nocturnal periods. For the conventional measurements, uncontrolled hypertension was defined as the achievement of values ≥ 140/90 mmHg, according to the main hypertension guidelines. For the group of patients aged ≥ 60 years, guidelines from the Eighth Joint National Committee (JNC 8) were also adopted.^1^

### Parameters and classification

In order to classify hypertension as uncontrolled, the ABPM criteria of the European Hypertension Guidelines and the Brazilian Hypertension Guidelines from the Brazilian Society of Cardiology were adopted.^12,13^ Thus, patients with a mean BP of ≥ 130/80 mmHg in 24 hours, ≥ 135/85 mmHg in the waking period and ≥ 120/70 mmHg for the nocturnal mean BP for the first criterion were considered as having uncontrolled hypertension.^12^ Whereas, when the guidelines of the Brazilian Guidelines for Hypertension were observed,^13^ the values for BP used in the study were the borderline ones considered normal as cutoff point for the 24-hour averages: >125/75 mmHg, > 130/85 mmHg for the waking period, and > 110/70 mmHg for the mean BP during sleep.^14^

A ≤ 10% reduction in the mean nocturnal BP in relation to the daytime mean in the ABPM was defined as an absence of nocturnal descent.^3^ The white coat syndrome (WCS) was considered to be present when a patient on antihypertensive treatment had high BP, as measured in the clinic environment and/or under surveillance, but controlled BP in other situations.^15^ Masked hypertension (MH) was characterized by the presence of a BP that was controlled when obtained with a conventional measurement, but high when obtained with ABPM or in-home measurements.^16^ "Masking effect" was the term used when MH was observed in hypertensive patients using antihypertensive treatment. The same values for the normality criteria for 24-hour BP were considered for diabetic and nondiabetic patients.

### Laboratory evaluation

In addition to BP measurements by the conventional method and the 24-hour ABPM, the biochemical profile of the patients in this study was evaluated. Laboratory tests included total cholesterol, LDL-cholesterol, HDL-cholesterol, triglycerides, creatinine, blood count, glycosylated hemoglobin (fraction A1c), microalbuminuria, and fasting blood glucose. Anthropometric data, such as body mass, height, waist-hip ratio, and body mass index were also evaluated. The screening questionnaire also included validated instruments for the evaluation of smoking and nicotine dependence (Fagerström test), abusive alcohol consumption (Alcohol Use Disorders Identification Test, AUDIT), and adherence to treatment using the Morisky Medication Adherence Scale.^8^

### Statistical methods

Data entry and analysis were performed using the SPSS statistical software, version 21.0. Descriptive statistics were performed with continuous variables (mean and standard deviation) and categorical variables (frequency distribution). The estimated sample size was 398 patients and was based on the previous cross-sectional study conducted at the same site of the current study and on BP control rates of 55% for ABPM and 41% for conventional measurements performed by the PHC physicians for a confidence interval of 95% (95%CI) and 80% power. The sample was considered representative of the PHC service in the city of Antônio Prado (RS) because it was randomly selected at the two health posts with hypertension outpatient clinics, out of a total of 1,216 patients enrolled in this system. The hypertension care units, where the study was conducted, are referential units of the PHC in the city.

The comparison between the subgroups was performed using the chi-square test, Mann-Whitney U test (for continuous variables with non-homogeneous variances), and Student's *t* test (for variables with homogeneous variances). To analyze the agreement between the techniques of BP evaluation, kappa statistics was used. A multivariate analysis was also performed for cardiovascular risk factors and agreement of BP control, according to the 24-hour ABPM compared with conventional BP. P values < 0.05 were considered significant.

## Results

Between January 2013 and October 2014, a consecutive sample of 639 hypertensive patients enrolled in the hypertension outpatient clinic of two health posts in the city of Antônio Prado (RS) was selected from a total of 1,216 patients. ABPM was applied in 611 patients who remained in the study after application of the exclusion criteria, shortly after conventional BP measurements by PHC physicians. The final sample comprised 569 patients after exclusion of patients who abandoned the research protocol or who presented inadequate ABPM measurements. No patient required medical care due to the compressive action of the ABPM cuff, whose reported events included: local discomfort (22 patients), mild local erythema (6 patients), and short-term paresthesia in the limb used for placing the cuff (2 patients). Table 1 summarizes the demographic profile and lifestyle of the patients.

**Table 1:** Demographic profile and lifestyle of the patients in the sample

	Total (n = 569)*
- Female n (%)	339 (59.6%)
- Age (years)	60.32 +/- 13.58 (20-89)
- White	504 (88.1%)
- BMI **	28.54 +/- 4.57 (19-46)
- Fasting blood glucose (mg/dL)	100 +/- 26.89 (47-279)
- Glycosylated hemoglobin	6.14 +/- 4.52 (2.7–11.2)
- Total cholesterol (mg/dL)	203.9 +/- 40.28 (88-453)
- HDL-cholesterol (mg/dL)	52.73 +/- 12.51 (39.1-66.2)
- LDL-cholesterol (mg/dL)	121.36 +/- 33.78 (14-241)
- Triglycerides (mg/dL)	155.98 +/- 136.55 (35-2446)
- Creatinine (mg/dL)	0.88 +/- 0.27 (0.41-3.09)
- Smoking n (%)	32 (5.6%)
- Alcohol consumption n (%) (> 30 g/day)	174 (30.6%)
- Microalbuminuria > 30 mg/dL n (%)	151 (26.4%)
- Physical activity n (%)	276 (48.5%)
- Physical activity > 150 min/week n (%)	148 (53.6%)

* Standard deviation, percentage and maximum and minimum values;

** body mass index (kg/m2).

In relation to BP measured by the conventional method *versus* 24-hour ABPM, we observed an accuracy of 54.8% (95%CI 0.51 - 0.58%) and, when considering the European Society of Hypertension Guidelines, a sensitivity of 85% (95%CI 80.8 - 88.6%), a specificity of 31.9% (95%CI 28.7 - 34.7%), and a kappa value of 0.155. When more stringent thresholds were used to characterize BP as "normal" by ABPM, we identified an accuracy of 45% (95%CI 0.41 - 0.47%) by the conventional measurement when compared with 24-hour ABPM, in addition to a sensitivity of 86.7% (95%CI 0.81 - 0.91%), a specificity of 29% (95%CI 0.26 - 0.30%), and a kappa value of 0.103 (Table 2).

**Table 2:** General accuracy for conventional measurement of blood pressure (BP) according to the various normality thresholds for 24-hour ambulatory blood pressure monitoring (ABPM) *

	Accuracy	Sensitivity	Specificity	95%C	kappa
ABPM 130/80 mmHg* X BP Conventional Method	54.8%	85%	31.9%	0.513-0.580	0.155
ABPM 125/75 mmHg X BP Conventional Method	45%	86.7%	29%	0.418 – 0.475	0.103
ABPM 130/80 mmHg* X BP JNC 8**	51.8%	88.5%	23.8%	0.485 – 0.546	0.111
ABPM 125/75 mmHg X BP JNC 8**	40.6%	89.8%	21.6%	0.377 – 0.428	0.072

* ESC 2013 and Joint 8

** SBP normal ≤ 150 mmHg. 95%CI: 95% confidence interval; JNC 8: Eighth Joint National Committee.

The prevalence of WCS and the masking effect in treated hypertensive patients was 3.1% and 46.9%, respectively, when considering the European Hypertension Guidelines for ABPM. On the other hand, the prevalence of WCS and MH was 2% and 56.1%, respectively, according to the different cutoff criteria for ABPM normality. When we considered the JNC 8 recommendations for conventional measurements, the prevalence of WCS and MH presented a slight variation. The prevalences according to the various parameters of ABPM and conventional measurement is shown in Table 3.

**Table 3:** White coat effect and masked hypertension according to the parameters of 24-hour ambulatory blood pressure monitoring (ABPM) and conventional measurement of blood pressure (BP)*

	White Coat Effect	Masked
ABPM 130/80 mmHg* X BP Conventional Method	6.5% (37)	38.7% (220)
ABPM 125/75 mmHg X BP Conventional Method	3.7% (21)	51.3% (292)
ABPM 130/80 mmHg * X BP JNC 8**	5% (28)	43.3% (244)
ABPM 125/75 mmHg X BP JNC 8**	2.8% (16)	56.6% (319)

* ESC 2013 e Joint 8

** PAS normal ≤ 150 mmHg. JNC 8: Eighth Joint National Committee.

## Discussion

The present study, including hypertensive patients receiving PHC, showed differences in accuracy and agreement between BP measurements performed by PHC physicians according to the different parameters of normality for 24-hour ABPM, as determined by the European Hypertension Guidelines and by the V Brazilian Guidelines for ABPM, respectively. The main result was the low accuracy of the conventional measures when compared with those obtained by the 24-hour ABPM, regardless of the guideline or cutoff point adopted for normal 24-hour ABPM. Regarding the differences in normality thresholds for 24-hour ABPM, when more stringent BP control targets were used, the accuracy of conventional measurements was even lower.

The vast majority of hypertensive patients are assisted by the PHC system,^17^ in which physicians play a relevant role in the search for better results in BP control. In addition, the use of auxiliary methods for BP measurements to assess the adequacy of antihypertensive treatment has not been widely adopted in PHC.^15^ In a national study using ABPM, a high degree of reproducibility was observed between casual measurements performed by non-medical professionals and those performed in an environment with a high standard of standardization in BP measurement.^18^ However, although casual BP measure is still the standard for hypertension diagnosis and control, its adoption with the rigor of controlled studies is not a reality in the current clinical practice of PHC. Additionally, the 24-hour BP assessment is the reference standard for prognostic evaluation, reduction of false diagnoses, and BP control evaluations.

In another national study with a retrospective analysis of ABPM examinations,^5^ the impact of the reclassification of BP control thresholds was evaluated according to the application of the last two Brazilian ABPM Guidelines. With the adoption of the current guidelines, all modified thresholds reclassified the exams significantly. The present study, however, sought to prospectively assess the impact of adopting different thresholds of normality for ABPM in comparison with measures performed by PHC physicians. The results, in terms of accuracy, were similar to those of other studies that indicated a low accuracy of BP obtained by conventional measures compared with that obtained by ABPM,^8^ but with unique results when comparing different guidelines for 24-hour mean pressure thresholds.

Current guidelines for hypertension recommend satisfactory BP control for cardiovascular protection in both primary and secondary prevention.^19^ The extent of agreement between the classification of controlled and uncontrolled BP, based on conventional measures and compared with 24-hour ABPM, is of strategic importance in PHC. The search for normality thresholds for 24-hour ABPM is based on cardiovascular outcomes,^14^ having the IDACO study database as an example of a population benchmark. These results guided the normality targets for ABPM in the different guidelines for hypertension. Thus, the adoption of normality criteria for ABPM as a gold-standard auxiliary method for the control, evaluation and prognostic stratification of hypertension^4^ should ideally be supported by a database that reflects population specificities.

The Systolic Blood Pressure Intervention Trial (SPRINT) study^20^ presents evidence of benefits for BP control in hypertensive patients with stringent measures for systolic pressure (< 120 mmHg) compared with a more flexible control (< 140 mmHg). Therefore, evidence of reduced outcomes with more stringent cutoff points for BP control may lead to substantial changes in future revisions of hypertension guidelines.^20^ Thus, this need is in agreement with the evaluation of the impact on the diagnostic accuracy of usual methods of BP in comparison with the different thresholds of normality for the gold-standard BP evaluation.

The adoption of the 24-hour ABPM through a single measurement in our sample may be considered a limitation of the present study. This may imply a limitation in the reproducibility of the measurements, especially when considering the evaluation of the nocturnal decreasing BP pattern. However, to mitigate this potential limitation, precautions were taken, such as the individualization of the sleep and waking period, as well as a rigidity regarding the minimum number of measurements and the quality of the measurements during the 24 hours as criteria for inclusion in the study. These measurements followed the guidelines and recommendations of the Italian Society of Hypertension.^21^

The use of ABPM in PHC, as well as the impact of the reclassification of hypertension according to the different normality thresholds for 24-hour BP, may have implications for decision-making by PHC physicians. Thus, a more significant number of national studies may serve as a reference for the elaboration of future guidelines and indicate BP thresholds for therapeutic definition.

## Conclusion

This study evaluated the use of 24-hour ABPM within the scope of the PHC. BP measurements assessed by PHC physicians presented low accuracy when compared with those obtained by 24-hour ABPM, regardless of the threshold used as a normality criterion.
